# The thermogenic effect of mirabegron ingestion during cool conditions

**DOI:** 10.3389/fphys.2025.1645475

**Published:** 2025-09-10

**Authors:** Felipe Gorini Pereira, Caleb T. Ryan, Spencer Miller, Andrew Watters, Timothy D. Mickleborough, Zachary J. Schlader, Christopher Bell, Blair D. Johnson

**Affiliations:** ^1^ Department of Kinesiology, School of Public Health, Indiana University, Bloomington, IN, United States; ^2^ Department of Emergency Medicine, Indiana University, Bloomington, IN, United States; ^3^ Department of Health & Exercise Science, Colorado State University, Fort Collins, CO, United States

**Keywords:** cold exposure, brown adipose tissue, beta-3 adrenergic receptor agonist, infrared thermography, mirabegron

## Abstract

**Introduction:**

Brown adipose tissue (BAT) is highly thermogenic and can be stimulated by cold exposure or mirabegron, a β3-adrenergic receptor agonist. However, it is currently not known whether the thermogenic effects of mirabegron are observed during exposure to cool temperatures. We tested the hypotheses that energy expenditure and BAT activation would be greater following mirabegron ingestion versus a placebo (PLA).

**Methods:**

Eleven healthy adults (5 women) completed four, double-blind, randomized visits to the laboratory involving the acute ingestion of 100, 150, and 200 mg of mirabegron or PLA. Following ingestion, subjects rested for 6 h in a whole-body indirect room calorimeter (20 °C, 50% RH). Cumulative energy expenditure was calculated for each study visit. Using infrared thermography, supraclavicular BAT activity was assessed via the area under the curve (AUC) for supraclavicular skin temperature (Tsc) expressed relative to sternal skin temperature for each visit.

**Results:**

Cumulative energy expenditure was greater following ingestion of 100 mg (494 ± 75 kcal; *p* < 0.001), 150 mg (481 ± 67 kcal; *p* = 0.017), and 200 mg (492 ± 69 kcal; *p* = 0.001) of mirabegron vs. PLA (456 ± 67 kcal), with no differences between doses (*p* > 0.05). The AUC for Tsc was greater following ingestion of 100 mg (12.54 ± 7.51 °C × min; *p* = 0.011) and 150 mg (10.01 ± 6.05 °C × min; *p* = 0.021) doses of mirabegron vs. PLA (4.74 ± 3.86 ° × min), but not the 200 mg dose (9.17 ± 5.95 ° × min; *p* = 0.067).

**Discussion:**

Our data indicate β3-adrenergic receptor activation of supraclavicular BAT via mirabegron ingestion enhances thermogenesis in cool environments.

## Introduction

Until recently, human brown adipose tissue (BAT) was thought to contribute to non-shivering thermogenesis (NST) only in infants (Aherne and Hull, 1966; Ito and Kuroshima, 1967). However, recent evidence (i.e., the last 15 years) demonstrates that BAT is present and contributes to energy expenditure and NST when activated in adult humans as well (Virtanen et al., 2009; Cypess et al., 2009; van Marken Lichtenbelt et al., 2009). Uncoupling protein 1 is expressed in BAT and contributes to NST by dissipating the proton gradient across the inner mitochondrial matrix, thus uncoupling mitochondrial respiration and resulting in heat production (Lowell et al., 1993). In this regard, the thermogenic potential of BAT may aid thermoregulation and thermal comfort during exposure to cold environments due to its contribution to NST (van der Lans et al., 2013).

BAT is sympathetically innervated and has a high density of β3-adrenergic receptors (Ursino et al., 2009). Cold-induced sympathetic excitation (Virtanen et al., 2009; Cypess et al., 2009) or the stimulation of β3-adrenergic receptors via pharmacological agents activates BAT and results in heat production (i.e., thermogenesis) (Cypess et al., 2015). Mirabegron (Myrbetriq, Astellas Pharma) is a β3-adrenergic receptor agonist approved by the U.S. Food and Drug Administration for the treatment of overactive bladder (Dehvari et al., 2018) and it stimulates β3-adrenergic receptors on BAT (Malik et al., 2012; Takasu et al., 2007). Recent evidence in humans indicates that acute doses of 100 mg–200 mg of mirabegron increases resting energy expenditure and elevates supraclavicular skin temperature (T_sc_), which is an indirect and noninvasive marker of BAT activation (Law et al., 2018a; Law et al., 2018b), for up to 180 min in a thermoneutral environment (Loh et al., 2019). Interestingly, Loh et al. observed increases in energy expenditure following the consumption of 100 and 200 mg doses of mirabegron, whereas increases in supraclavicular skin temperature were only observed following the consumption of 100 and 150 mg doses of mirabegron (Loh et al., 2019). Furthermore, utilizing position emission tomography alongside computed tomography (PET/CT) techniques, Cypess et al. found that the acute ingestion of 200 mg of mirabegron increased the metabolic activity of BAT in healthy adults, which was measured via ^18^F-fluorodeoxyglucose uptake, in a 23 °C testing environment (Cypess et al., 2015).

Due to its thermogenic effects, the activation of BAT via mirabegron could aid thermoregulation. However, it is unclear whether the effects of mirabegron on BAT activation and energy expenditure extend beyond thermoneutral conditions (i.e., air temperature 23 °C–26 °C) (Cypess et al., 2015; Loh et al., 2019). Given that BAT can be independently activated by exposure to cool environments (Virtanen et al., 2009; Cypess et al., 2009) or the pharmacological stimulation of β3-adrenergic receptors (Cypess et al., 2015), the extent to which mirabegron modulates BAT activation during exposure to cool ambient temperatures is not known. Moreover, it is unknown whether the metabolic effects of an acute dose of mirabegron extend beyond 4 h. Pharmacological data suggest that plasma concentrations of mirabegron peak between 3 and 4 h following administration (Malik et al., 2012), whereas its biological half-life is approximately 50 h (Eltink et al., 2012). Thus, the thermogenic effects of mirabegron might extend beyond the previously studied timelines (i.e., up to 4 h). Therefore, the purpose of our study was to determine whether energy expenditure and T_sc_ (i.e., noninvasive marker of supraclavicular BAT activation) during 6 h of rest in a moderately cool environment (i.e., 20 °C) are greater following acute doses of mirabegron (100, 150, and 200 mg) versus a placebo (PLA). We hypothesized the following: (1) energy expenditure and T_sc_ following each dose of mirabegron would be greater than PLA, and (2) 100 and 200 mg of mirabegron would elicit the greatest resting energy expenditure.

## Methods

### Participants

**FIGURE 1:**
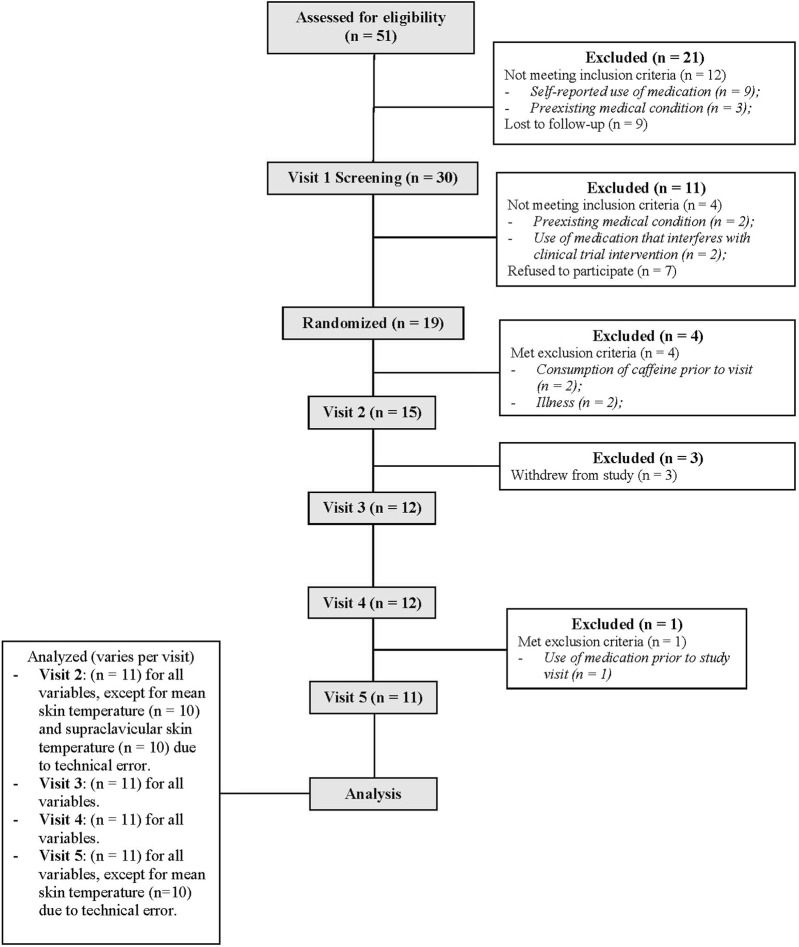
CONSORT flow diagram illustrating the recruitment process throughout our randomized, double-blind, placebo-controlled, cross-over clinical trial.

Eleven healthy adults (five women) participated in the study (Table 1). The study included one informed consent and screening visit, followed by four experimental visits. In accordance with our exclusion criteria, all participants self-reported to be nonsmokers and free from cardiovascular, metabolic, neurologic, gastrointestinal, and respiratory diseases. All participants were also medically screened by our study physician (A. Watters) prior to taking part in the study for any contraindications to mirabegron ingestion (CONSORT recruitment diagram shown in Figure 1). Participants were not taking any medication at the time of the study, except oral contraceptives (n = 2). Women were confirmed not to be pregnant through a negative urine pregnancy test prior to every study visit. The menstrual cycle phase was not controlled for in this study. For our female participants, thirteen experimental visits were conducted during the follicular phase (i.e., within 14 days of previous menstruation) and seven visits were conducted in the luteal phase (i.e., more than 14 days from previous menstruation). The study staff verbally explained the research procedures and the potential risks to each participant. Participants then provided written informed consent in accordance with the latest version of the Declaration of Helsinki. The study was registered at clinicaltrials.gov (NCT04766021). All procedures were approved by the Institutional Review Board at Indiana University.

**TABLE 1:** Subject demographics.

Variables	Mean ± SD
Age (years)	22 ± 2
Height (cm)	172.8 ± 17.5
Weight (kg)	78.0 ± 13.4
Body mass index (kg/m^2^)	25.3 ± 4.5
Body fat (%)	23.0 ± 4.6

Data are represented as the mean ± SD. n = 11 (5 females).

### Experimental design

We employed a randomized, double-blind, placebo-controlled, crossover research design. Following the first visit, demographic and anthropometric data were collected (i.e., height, nude body weight, heart rate, blood pressure, and body composition *via* dual energy X-ray absorptiometry), and participants completed a medical history questionnaire. For the experimental visits, participants reported to the temperature-controlled laboratory (25 °C ± 1 °C) following a 12-h fast and after abstaining from alcohol, caffeine, and strenuous exercise for 24 h. Mirabegron has a half-life of approximately 50 h (Dehvari et al., 2018); thus, all experimental visits were separated by a minimum of 10 days. To account for the effects of the diet, participants were provided a diet log to complete 72 h prior to the first experimental visit and were instructed to replicate their food intake for the subsequent experimental visits. Food and fluid intake data from the diet logs were analyzed for the total caloric intake along with carbohydrate, protein, and fat intake using an online software application (MyFitnessPal). Data from these dietary logs are presented in Table 2.

**TABLE 2:** Dietary assessment.

Variables	PLA	100 mg	150 mg	200 mg	p-value
Caloric intake (kcal)	5,297 ± 1,624	5,514 ± 1,531	5,589 ± 1,566	5,427 ± 1,616	0.869
Carbohydrate intake (g)	579 ± 202	618 ± 208	667 ± 230	559 ± 149	0.420
Protein intake (g)	227 ± 66	248 ± 88	264 ± 91	246 ± 113	0.575
Fat intake (g)	314 ± 67	211 ± 76	227 ± 90	221 ± 59	0.906

Data are represented as the mean ± SD. Data for dietary assessment were analyzed using mixed-effects models (i.e., dose × time). n = 11 for all comparisons (5 females).

Upon arrival to the laboratory, participants provided a urine sample for hydration assessment. Adequate hydration was defined as a urine specific gravity (USG) < 1.025 (Armstrong et al., 1994). Participants then measured their nude body weight and were instructed to self-insert a rectal thermistor probe in a private room. Participants consumed a standard-light breakfast (237 mL of Ensure Original; 220 kcal) and rested quietly in a seated position during instrumentation. Following 20 min of quiet rest, 5 min of baseline data (i.e., heart rate and blood pressure) were acquired prior to drug administration. Participants then ingested mirabegron (100, 150, or 200 mg) (Myrbetriq; Astellas Pharma US) or PLA (anhydrous lactose powder in gelatin capsules) with 250 mL of water. Each 50 mg mirabegron tablet was placed within a gelatin capsule to ensure that the participants and study personnel were blinded to the treatment condition. Participants ingested four capsules for each treatment condition that consisted of the following: four 50-mg mirabegron capsules (200 mg condition), three 50-mg mirabegron and one PLA capsule (150 mg condition), two 50-mg mirabegron capsules and two PLA capsules (100 mg condition), or four PLA capsules (PLA). After 30 min following mirabegron/PLA ingestion, a thermal image was acquired prior to the participants entering the whole-body indirect room calorimeter, wearing only shorts (for men) or shorts and a sports bra (for women). The chamber temperature was set at 20 °C and 50% relative humidity, eliciting a mild cool stress throughout the measurement period due to the minimal amount of clothing worn during experimental visits. This cool temperature was purposefully utilized in the current study design as it is above the human shivering threshold, allowing for the assessment of non-shivering thermogenesis while maintaining participant comfort during the measuring period. Participants rested in this cool environment in a semi-recumbent position for 6 h. All participants briefly exited the chamber to use the restroom halfway through the 6-h protocol. During the exposure, participants were instructed to remain sedentary. Participants watched a non-stimulating documentary or read. The study personnel logged the behavior (i.e., time spent reading or time spent watching a documentary) during the first experimental visit and instructed the participants to conduct the same behavior for the same duration for subsequent visits.

### Instrumentation and measurements

The height (cm) and nude body weight (kg) were measured using a stadiometer (Holtain Limited, Seritex, Wales, United Kingdom) and scale (Sauter, Balingen, Germany), and the body composition was measured via dual-energy X-ray absorptiometry (GE Lunar iDXA; Getz Healthcare, Lane Cove, NSW, Australia). Prior to all experimental visits, USG was measured using a handheld refractometer (Atago, Tokyo, Japan). A small (chamber volume = 4,300 L) whole-body indirect room calorimeter (MEI Research, Enida, MN) was utilized to assess and calculate oxygen consumption (VO_2_; L/min), carbon dioxide production (VCO_2_; L/min), and the respiratory exchange ratio (RER; VCO_2_/VO_2_) throughout the 6-h measurement period. T_sc_ was accessed via infrared thermography (FLIR T560, FLIR Systems AB, Danderyd, Sweden) as a noninvasive measurement of supraclavicular BAT activation (Law et al., 2018a; Law et al., 2018b). Relatively large deposits of BAT are superficially located in the supraclavicular fossa (Heaton, 1972). Thus, increases in T_sc_ are interpreted as increases in BAT metabolism (Law et al., 2018a; Law et al., 2018b; Haq et al., 2017). This measurement has been shown to be reproducible (Haq et al., 2017) and is highly correlated with ^18^F-fluorodeoxyglucose uptake measured via PET/CT, which is considered to be the gold standard for quantifying BAT activity (Law et al., 2018b). Images were captured from a constant distance of 1 m, and the participants wore identical clothing for all experimental visits.

Rectal temperature (T_rec_) was measured continuously as an index of core temperature using a thermistor probe (Covidien, Medtronic, Minneapolis, MN) that was self-inserted to a depth of 10 cm past the anal sphincter. The mean skin temperature (T_sk_), calculated using 12 sites (i.e., the forehead, chest, abdomen, subscapular, lower back, forearm, dorsal hand, anterior thigh, hamstring, shin, calf, and foot), was measured continuously using Thermochron iButtons (Maxim Integrated, San Jose, CA). Shivering was assessed via mechanomyography (MMG) using three separate tri-axial accelerometers, with movement recorded across the X-, Y-, and Z-axes (Arnold et al., 2020; Arnold et al., 2023). Subjective assessments of shivering were also obtained by two investigators every 30 min during the measurement period using a validated Likert scale (i.e., bedside shivering scale; 0 = no shivering and 3 = severe shivering) (Badjatia et al., 2008). The bedside shivering scale ratings were averaged and recorded as means for each timepoint. The heart rate was measured continuously using a 3-lead electrocardiogram (Datex-Ohmeda, Instrumentarium Corp, Helsinki, Finland). Blood pressure was measured at baseline and at every 30 min throughout the measurement period using an automated blood pressure cuff (Datex-Ohmeda, Instrumentarium Corp, Helsinki, Finland) on the left brachium. Participant thermal discomfort (1 = comfortable and 4 = very uncomfortable) and sensation (1 = cold, 4 = neutral, and 7 = hot) were obtained to assess thermal perceptions every 30 min (Gagge et al., 1967).

### Data analysis

Data were collected for every 30 min timepoint during the 6-h exposure. The VO_2,_ VCO_2_, and RER mean values were calculated for the last 10 min of each 30 min timepoint. Energy expenditure (kcal/min/kg^−1^) and cumulative resting energy expenditure (kcal) were calculated for each 6-h exposure utilizing the abbreviated Weir formula (Weir, 1949) as a method of assessing thermogenesis. Thermal images were obtained every 30 min and saved as JPEG files. Using the FLIR Studio Research software (FLIR T560, FLIR Systems AB, Danderyd, Sweden), two regions of interest (ROIs) were defined: the right supraclavicular region and a reference region (i.e., the sternal notch). Anatomical placement of both ROIs was defined according to the specifications previously suggested by Law et al. (2018a) and Law et al. (2018b). We then identified the hottest 10% of the pixels within each ROI using MATLAB (version 23.2, MathWorks Inc., Natick, MA), and the medians of these pixels were calculated as T_sc_ and sternal skin temperature. T_sc_ is expressed as both an absolute value and relative to sternal skin temperature (relative T_sc_ = absolute T_sc_ – sternal skin temperature). Additionally, the area under the curve (AUC) values for absolute and relative T_sc_ were calculated for each study visit. The AUC values for both absolute and relative T_sc_ were calculated based on changes from the pre-exposure thermal images. The mean T_sk_ was calculated as the weighted average of the 12 iButtons using the following equation: (0.07*forehead) + (0.14*forearm) + (0.5*dorsal hand) + (0.07*foot) + [0.13*(shin + calf)/2] + [0.19*(hamstring + anterior thigh)/2] + [0.35*(chest + abdomen + subscapular + lower back)/4] (Hardy et al., 1938). The mean body temperature was calculated using T_rec_ and mean T_sk_ values using the following equation: 0.64 * T_rec_ + 0.36 * T_sk_ (Burton, 1935). Accelerometers sampled the movement events at each site at a frequency of 100 Hz and were analyzed as an assessment of shivering using the root sum of the squares of each axis using R (R Foundation for Statistical Computing) (Arnold et al., 2020). The pulse pressure (systolic blood pressure – diastolic blood pressure) and mean arterial pressure (diastolic blood pressure + 1/3 * pulse pressure) were calculated. The rate pressure product (heart rate * systolic blood pressure) was also calculated as an estimate of myocardial oxygen demand.

### Statistical analysis

The sample size was determined with G*Power 3.1.9.4 (Faul et al., 2007). Based on resting energy expenditure and supraclavicular skin temperature data reported by Loh et al. (2019), we estimated an effect size (f) of 0.90 for the comparison between the placebo condition and mirabegron doses. Using this estimate, a power analysis determined that a sample size of ten participants would be sufficient to achieve a statistical power of 0.99, with the alpha level set at p ≤ 0.05. Data were analyzed using GraphPad Prism version 10.2.2 (Prism, San Diego, CA) statistical software. Normality of the dependent variables was assessed using the Shapiro–Wilk test. All dependent variables collected at baseline and following the ingestion of 100, 150, and 200 mg of mirabegron or PLA were analyzed using mixed-effects models (i.e., dose × time). If a significant dose effect was found, the Bonferroni *post hoc* procedure was used to determine the conditions that differed. Cumulative resting energy expenditure and the AUC for absolute and relative T_sc_ and sternal skin temperature were analyzed using mixed-effects models, and if a significant condition effect was found, the Bonferroni *post hoc* procedure was used to determine the conditions that differed (Lee and Lee, 2018). An alpha level of 0.05 was set *a priori* to denote the statistical significance for all comparisons. For all significant effects, effect sizes were calculated as partial eta squared (η_p_^2^) to provide the reader with an indication of the magnitude of the observed differences (Cohen, 1988). For reference values, 0.01, 0.09, and 0.25 are considered to be small, medium, and large effect sizes for η_p_^2^, respectively. Data are presented as the mean ± standard deviations along with individual values, where appropriate.

## Results

### Metabolic outcomes

#### Oxygen consumption

**FIGURE 2:**
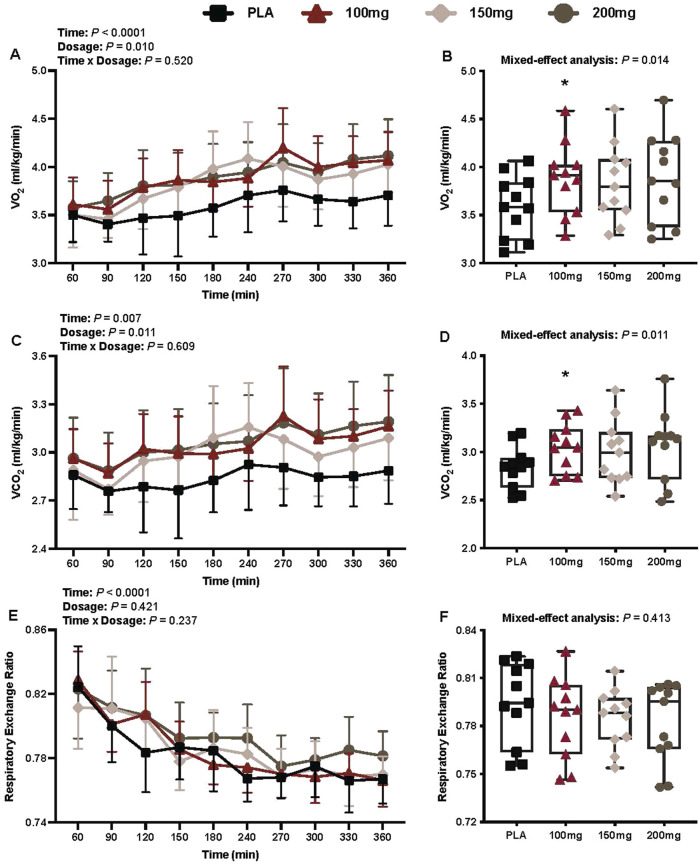
VO_2_ **(A)**, mean VO_2_ per condition **(B)**, VCO_2_ **(C)**, mean VCO_2_ per condition **(D)**, RER **(E)**, and mean RER per condition **(F)** are shown for all the visits. Data are expressed as the mean ± standard deviations and individual values. Data for VO_2_, VCO_2_, and RER were analyzed using mixed-effects models (i.e., dose × time). Mean values per condition were analyzed using mixed-effects models, and if a significant condition effect was found, the Bonferroni *post hoc* procedure was used to determine the conditions that differed. n = 11 for all comparisons (five females).*= different from PLA (*p* < 0.050).

There was a main effect of time (*p* < 0.001; η_p_^2^ = 0.57) and dosage (*p* = 0.010; η_p_^2^ = 0.35) for VO_2_ (Figure 2A). VO_2_ was greater following the ingestion of the 100 mg (*p* = 0.014; η_p_^2^ = 0.61) dose of mirabegron versus PLA (Figure 2B).

#### Carbon dioxide production

There was a main effect of time (*p* = 0.007; η_p_^2^ = 0.34) and dosage (*p* = 0.011; η_p_^2^ = 0.36) for VCO_2_ (Figure 2C). VCO_2_ was greater following the ingestion of the 100 mg (p = 0.007; η_p_^2^ = 0.66) dose of mirabegron versus PLA (Figure 2D).

#### Respiratory exchange ratio

There was a main effect of time for RER (*p* < 0.001; η_p_^2^ = 0.71; Figure 2E). There was no interaction (*p* = 0.237) or effect of dosage (*p* = 0.421) for RER (Figure 2E).

#### Energy expenditure

**FIGURE 3:**
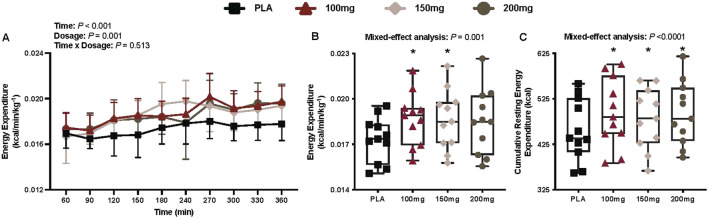
Energy expenditure **(A)**, mean energy expenditure per condition **(B)**, and cumulative resting energy expenditure per condition **(C)** are shown for all the visits. Data are expressed as the mean ± standard deviations and individual values. Data for energy expenditure were analyzed using mixed-effects models (i.e., dose × time). Mean values per condition were analyzed using mixed-effects models, and if a significant condition effect was found, the Bonferroni *post hoc* procedure was used to determine the conditions that differed. n = 11 for all comparisons (five females). * = different from PLA (p < 0.050).

There was a main effect of time (*p* < 0.001; η_p_^2^ = 0.48) and dosage (*p* = 0.001; η_p_^2^ = 0.41) for energy expenditure (Figure 3A). Energy expenditure was greater following the ingestion of the 100 mg (*p* = 0.012; η_p_^2^ = 0.63) and 150 mg (*p* = 0.041; η_p_^2^ = 0.54) doses of mirabegron versus PLA (Figure 3B).

#### Cumulative resting energy expenditure

Compared to PLA, 11 participants had greater cumulative resting energy expenditure in the 100 mg condition, nine in the 150 mg condition, and 11 in the 200 mg condition. There was a main effect of dose for cumulative resting energy expenditure (*p* < 0.001; η_p_^2^ = 0.54; Figure 3C). Cumulative resting energy expenditure was greater following 100 mg (1,978 ± 288.8 kcal; %Δ from PLA = 8.39 ± 3.96%, *p <* 0.001; η_p_^2^ = 0.81), 150 mg (1,925 ± 247.6 kcal; %Δ from PLA = 5.68 ± 5.24%, *p* = 0.039; η_p_^2^ = 0.54), and 200 mg (1,978 ± 266.1 kcal; %Δ from PLA = 8.21 ± 5.07%, *p* = 0.002; η_p_^2^ = 0.73) doses of mirabegron versus PLA (1,825.4 ± 258.5 kcal).

### Infrared thermography outcomes

#### Absolute supraclavicular temperature

**FIGURE 4:**
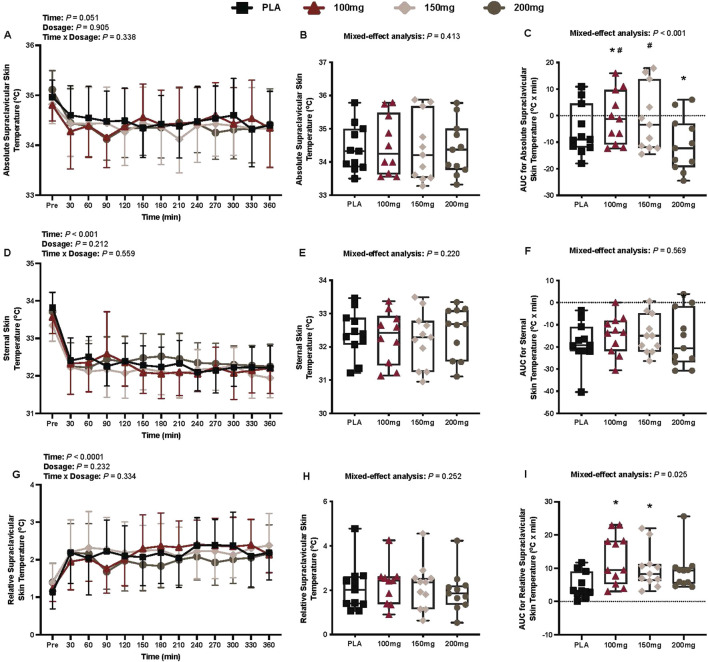
Absolute supraclavicular skin temperature **(A)**, mean absolute supraclavicular skin temperature per condition **(B)**, AUC for absolute supraclavicular skin temperature **(C)**, sternal skin temperature **(D)**, mean sternal skin temperature per condition **(E)**, AUC for sternal skin temperature **(F)**, relative supraclavicular skin temperature **(G)**, mean relative supraclavicular skin temperature per condition **(H)**, and the AUC for relative supraclavicular skin temperature **(I)** are shown for all the visits. Data are expressed as mean ± standard deviations and individual values. Data for the absolute and relative supraclavicular skin temperature were analyzed using mixed-effects models (i.e., dose × time). Mean and AUC values per condition were analyzed using mixed-effects models, and if a significant condition effect was found, the Bonferroni *post hoc* procedure was used to determine the conditions that differed. n = 11 for all comparisons (five females). * = different from PLA (p < 0.050). **#** = different from the 200 mg dose (p < 0.050).

There was no interaction (*p* = 0.338), effect of dosage (*p* = 0.905), or time (*p* = 0.051) for absolute T_sc_ (Figure 4A).

#### AUC for absolute supraclavicular temperature

Compared to PLA, eight participants had a greater AUC for absolute T_sc_ in the 100 mg condition, eight in the 150 mg condition, and two in the 200 mg condition. There was a main effect of dose for the AUC for absolute T_sc_ (*p* < 0.001; η_p_^2^ = 0.70; Figure 4C). The AUC for absolute T_sc_ differed from PLA following the ingestion of 100 mg (*p* = 0.041; η_p_^2^ = 0.49) and 200 mg (*p* = 0.003; η_p_^2^ = 0.72) doses of mirabegron (Figure 4C). Similarly, the AUC for absolute T_sc_ differed between the 200 mg dose of mirabegron and 100 mg (*p* < 0.001; η_p_^2^ = 0.89) and 150 mg (*p* < 0.001; η_p_^2^ = 0.88) doses (Figure 4C).

#### Sternal skin temperature

There was a main effect of time for sternal skin temperature (*p* < 0.001; η_p_^2^ = 0.67; Figure 4D). There was no interaction (*p* = 0.559) or effect of dosage (*p* = 0.212) for sternal skin temperature (Figure 4D).

#### AUC for sternal skin temperature

There was no main effect of dose for the AUC for sternal skin temperature (*p* = 0.569; Figure 4F).

#### Relative supraclavicular temperature

There was a main effect of time for relative T_sc_ (*p* < 0.001; η_p_^2^ = 0.53; Figure 4G). There was no interaction (*p* = 0.334) or effect of dosage (*p* = 0.232) for relative T_sc_ (Figure 4G).

#### AUC for supraclavicular temperature

Compared to PLA, 10 participants had a greater AUC for relative T_sc_ in the 100 mg condition, 11 in the 150 mg condition, and nine in the 200 mg condition. There was a main effect of dose for the AUC for relative T_sc_ (*p* = 0.025; η_p_^2^ = 0.32; Figure 4I). The AUC for relative T_sc_ differed from PLA following the ingestion of 100 mg (*p* = 0.019; η_p_^2^ = 0.57) and 150 mg (*p* = 0.035; η_p_^2^ = 0.52) doses of mirabegron (Figure 4I).

### Thermal outcomes

#### Rectal temperature

**FIGURE 5:**
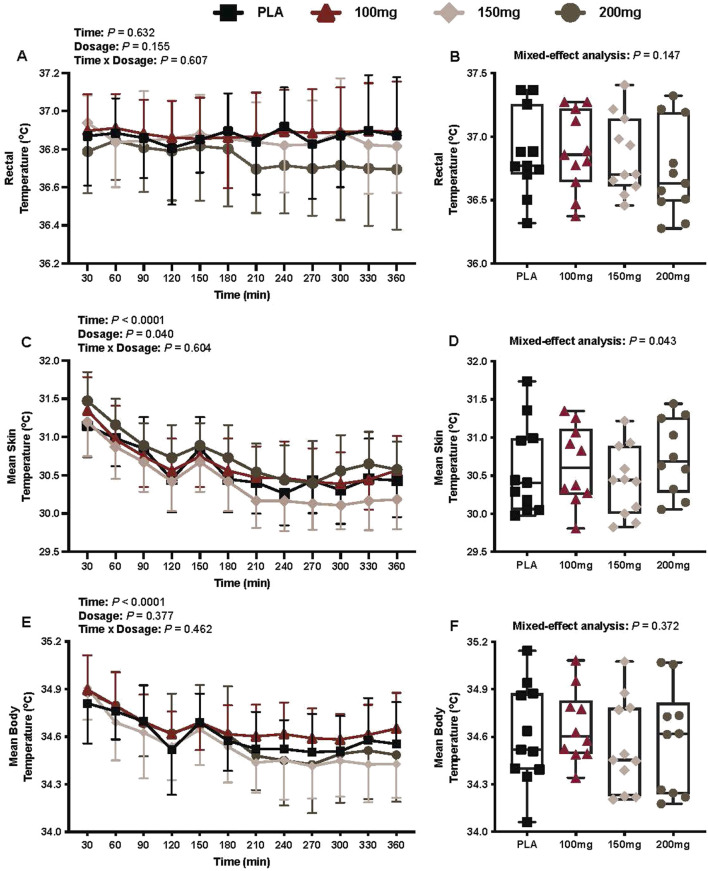
T_rec_ **(A)**, average T_rec_ per condition **(B)**, mean skin temperature **(C)**, average mean skin temperature per condition **(D)**, mean body temperature **(E)**, and average mean body temperature per condition **(F)** are shown for all the visits. Data are expressed as the mean ± standard deviations and individual values. Data for T_rec_, the mean skin temperature, and the mean body temperature were analyzed using mixed-effects models (i.e., dose × time). Averaged values per condition were analyzed using mixed-effects models, and if a significant condition effect was found, the Bonferroni *post hoc* procedure was used to determine the conditions that differed. n = 11 for all comparisons (five females). # = different from the 200 mg dose (p < 0.050).

There was no interaction (p = 0.607) or effect of dosage (p = 0.155) or time (p = 0.607) for T_rec_ (Figure 5A).

#### Mean skin temperature

There was a main effect of time (*p* < 0.001; η_p_^2^ = 0.69) and dosage (*p* = 0.040; η_p_^2^ = 0.26) for T_sk_ (Figure 5C).

#### Mean body temperature

There was a main effect of time for the mean body temperature (*p* < 0.001; η_p_^2^ = 0.62; Figure 5E). There was no interaction (*p* = 0.462) or effect of dosage (*p* = 0.377) for the mean body temperature (Figure 5E).

### Shivering activity

#### Mechanomyography

**FIGURE 6:**
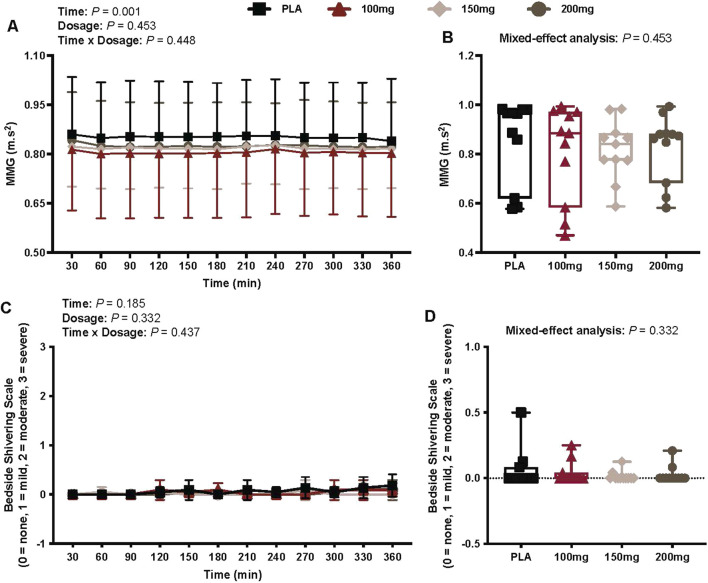
MMG **(A)**, mean MMG per condition **(B)**, bedside shivering scale ratings **(C)**, and mean bedside shivering scale ratings per condition **(D)** are shown for all the visits. Data are expressed as the mean ± standard deviations and individual values. Data for MMG and bedside shivering scale ratings were analyzed using mixed-effects models (i.e., dose × time). The mean values per condition were analyzed using mixed-effects models, and if a significant condition effect was found, the Bonferroni post hoc procedure was used to determine the conditions that differed. n = 11 for all comparisons (five females).

There was a main effect of time (*p* = 0.001; η_p_^2^ = 0.38) for MMG (Figure 6A). There was no interaction (*p* = 0.448) or effect of dosage (*p* = 0.453) for MMG (Figures 6A,B).

#### Bedside shivering scale

There was no interaction (*p* = 0.437) or effect of dosage (*p* = 0.332) and time (*p* = 0.185) for bedside shivering scale measures (Figure 6C).

### Cardiovascular outcomes

#### Heart rate

**FIGURE 7:**
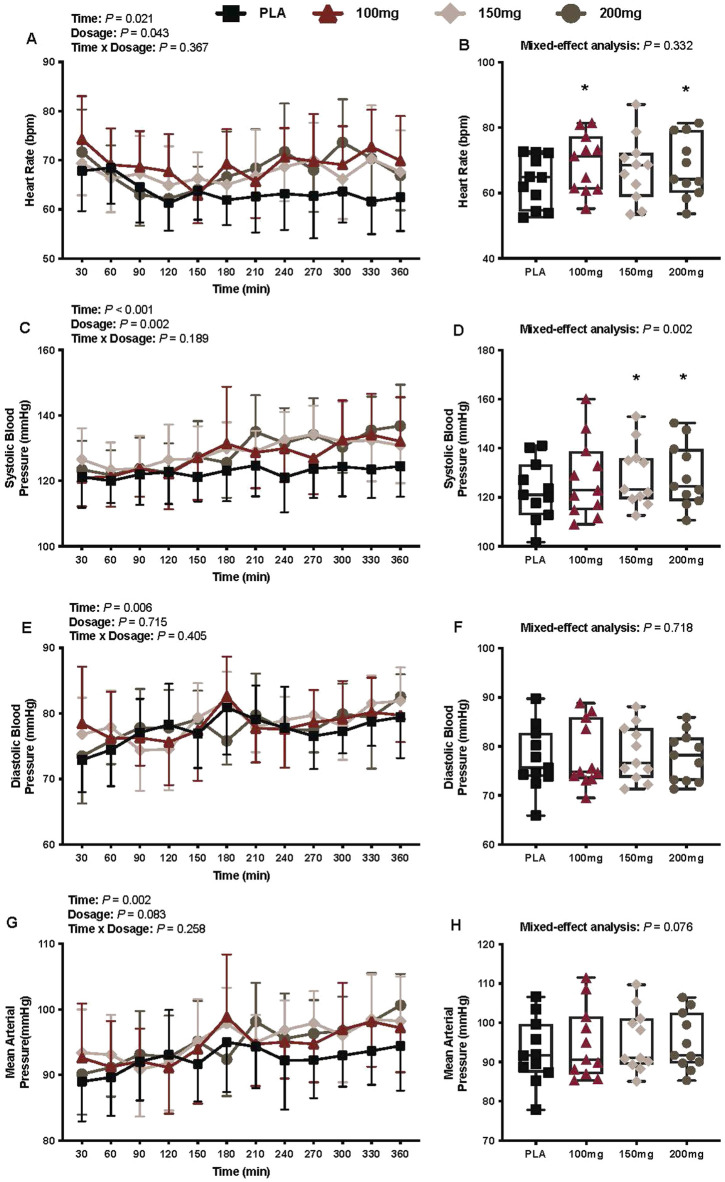
Heart rate **(A)**, mean heart rate per condition **(B)**, systolic blood pressure **(C)**, average systolic blood pressure per condition **(D)**, diastolic blood pressure **(E)**, average diastolic blood pressure per condition **(F)**, mean arterial pressure **(G)**, and average mean arterial pressure per condition **(H)** are shown for all the visits. Data are expressed as the mean ± standard deviations and individual values. Data for heart rate, systolic blood pressure, diastolic blood pressure, and mean arterial pressure were analyzed using mixed-effects models (i.e., dose × time). Averaged values per condition were analyzed using mixed-effects models, and if a significant condition effect was found, the Bonferroni *post hoc* procedure was used to determine the conditions that differed. n = 11 for all comparisons (five females). * = different from PLA (p < 0.050).

There was a main effect of time (*p* = 0.021; η_p_^2^ = 0.24) and dosage (*p* = 0.043; η_p_^2^ = 0.26) for the heart rate (Figure 7A). The heart rate was greater following the ingestion of 100 mg (*p* = 0.049; η_p_^2^ = 0.52) and 200 mg (*p* = 0.015; η_p_^2^ = 0.61) doses of mirabegron versus PLA (Figure 7B).

#### Systolic blood pressure

There was a main effect of time (*p* < 0.001; η_p_^2^ = 0.43) and dosage (*p* = 0.002; η_p_^2^ = 0.41) for systolic blood pressure (Figure 7C). The systolic blood pressure was greater following the ingestion of 150 mg (*p* = 0.014; η_p_^2^ = 0.62) and 200 mg (*p* = 0.002; η_p_^2^ = 0.73) doses of mirabegron versus PLA (Figure 7D)

#### Diastolic blood pressure

There was a main effect of time (*p* = 0.006; η_p_^2^ = 0.28) for diastolic blood pressure (Figure 7E). There was no interaction (*p* = 0.405) or effect of dosage (*p* = 0.715) for diastolic blood pressure (Figure 7E).

#### Mean arterial pressure

There was a main effect of time (*p* = 0.002; η_p_^2^ = 0.42) for the mean arterial pressure (Figure 7G). There was no interaction (*p* = 0.258) or effect of dosage (*p* = 0.083) for mean arterial pressure (Figure 7G).

#### Pulse pressure

**FIGURE 8:**
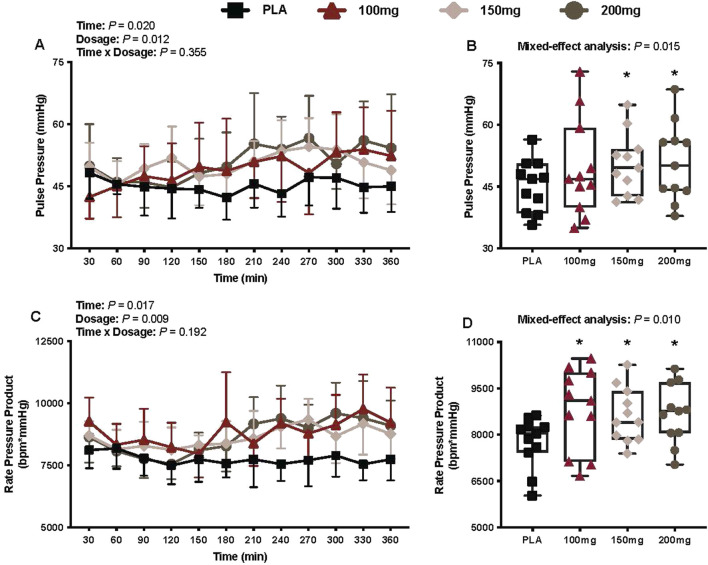
Pulse pressure **(A)**, mean pulse pressure per condition **(B)**, rate pressure product **(C)**, and mean rate pressure product per condition **(D)** are shown for all the visits. Data are expressed as the mean ± standard deviations and individual values. Data for pulse pressure and rate pressure product were analyzed using mixed-effects models (i.e., dose × time). Averaged values per condition were analyzed using mixed-effects models, and if a significant condition effect was found, the Bonferroni *post hoc* procedure was used to determine the conditions that differed. n = 11 for all comparisons (five females). * = different from PLA (p < 0.050).

There was a main effect of time (*p* = 0.020; η_p_^2^ = 0.23) and dosage (*p* = 0.012; η_p_^2^ = 0.33) for pulse pressure (Figure 8A). The pulse pressure was greater following the ingestion of the 150 mg (*p* = 0.010; η_p_^2^ = 0.64) and 200 mg (*p* = 0.011; η_p_^2^ = 0.63) doses of mirabegron versus PLA (Figure 8B).

#### Rate pressure product

There was a main effect of time (*p* = 0.017; η_p_^2^ = 0.27) and dosage (*p* = 0.009; η_p_^2^ = 0.39) for the rate pressure product (Figure 8C). The rate pressure product was greater following the ingestion of 100 mg (*p* = 0.037; η_p_^2^ = 0.54), 150 mg (*p* = 0.018; η_p_^2^ = 0.60), and 200 mg (*p* = 0.002; η_p_^2^ = 0.84) doses of mirabegron versus PLA (Figure 8D).

### Perceptual outcomes

#### Thermal discomfort

**FIGURE 9:**
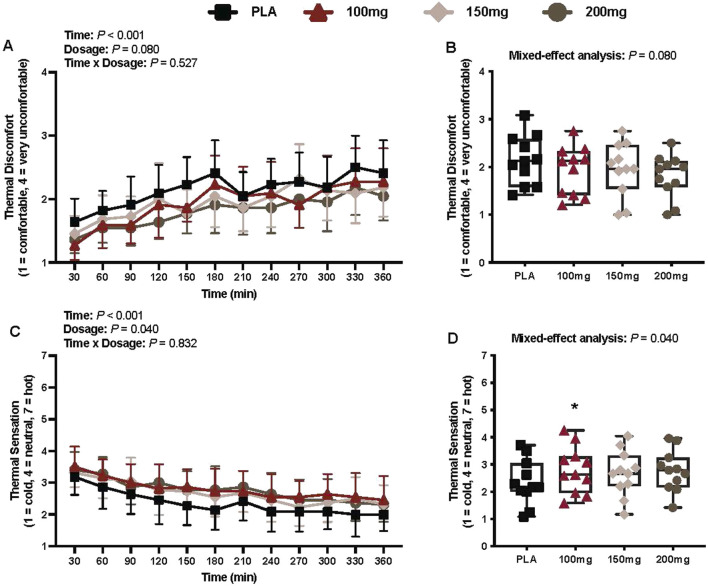
Thermal discomfort **(A)**, mean thermal discomfort per condition **(B)**, thermal sensation **(C)**, and mean thermal sensation per condition **(D)** are shown for all the visits. Data are expressed as the mean ± standard deviations and individual values. Data for thermal discomfort and sensation were analyzed using mixed-effects models (i.e., dose × time). Averaged values per condition were analyzed using mixed-effects models, and if a significant condition effect was found, the Bonferroni *post hoc* procedure was used to determine the conditions that differed. n = 11 for all comparisons (five females). * = different from PLA (p < 0.050).

There was a main effect of time (*p* < 0.001; η_p_^2^ = 0.57) for thermal discomfort (Figure 9A). There was no interaction (*p* = 0.527) or effect of dosage (*p* = 0.080) for thermal discomfort (Figure 9A).

#### Thermal sensation

There was a main effect of time (*p* < 0.001; η_p_^2^ = 0.62) and dosage (*p* = 0.040; η_p_^2^ = 0.29) for thermal sensation (Figure 9C). Thermal sensation was greater following the ingestion of the 100 mg (*p* = 0.027; η_p_^2^ = 0.57) dose of mirabegron versus PLA (Figure 9D).

## Discussion

The primary findings from our study are as follows: (1) in accordance with our first hypothesis, acute doses of mirabegron (i.e., 100, 150, and 200 mg) elicited greater energy expenditure (i.e., cumulative resting energy expenditure; Figure 3C) and higher T_sc_, with the exception of the 200 mg dose (i.e., Figure 4I) versus PLA ingestion, and (2) contrary to our second hypothesis, there were no observed differences in energy expenditure (Figure 3B) and or T_sc_ (Figures 4B,H) among the mirabegron doses. Furthermore, mirabegron ingestion did not affect T_rec_ (Figure 5A) or the mean body temperature (Figure 5E) during a 6-h exposure in a cool environment despite an observed improvement in thermal sensation ratings following the ingestion of the 100 mg dose of mirabegron (Figure 9D). Although our metabolic and infrared thermography data align with previous investigations, our experimental design was unique in several ways: (1) we utilized a whole-body indirect room calorimeter to assess changes in metabolism, (2) we observed responses for 6 h, (3) participants were exposed to a cool environment, and (4) we simultaneously measured supraclavicular BAT activation via infrared thermography, core and skin temperatures, cardiovascular responses, and thermal perceptions throughout the exposures.

Our findings support our first hypothesis that acute mirabegron ingestion increases resting energy expenditure and elevates the supraclavicular skin temperature, likely due to enhanced supraclavicular BAT activation, during exposure to a cool environment. Our findings align with published data by other investigators where the experiments were conducted in thermoneutral environments (Cypess et al., 2015; Loh et al., 2019; O'Mara et al., 2020). Acute mirabegron ingestion, regardless of the dose, elicited a 5.1%–8.4% increase in resting energy expenditure (Figure 3C) during the 6-h exposure to a cool environment in our study. The magnitude of the observed changes in resting energy expenditure is consistent with that reported in previous investigations. Cypess et al. (2015) observed a 13% increase in energy expenditure following the ingestion of 200 mg of mirabegron. Similarly, O'Mara et al. (2020) reported a 10.7% increase in resting energy expenditure following the ingestion of 100 mg of mirabegron. The cool environment utilized in our study increased thermogenesis over time (Figure 3A), which likely explains the slightly attenuated increases in resting energy expenditure (i.e., mirabegron vs. PLA conditions) compared to findings from other investigations. Collectively, our data and findings from these previous investigations indicate that the acute ingestion of 100 mg–200 mg of mirabegron augments whole-body energy expenditure. Moreover, as all mirabegron doses elicited an increase in energy expenditure versus PLA ingestion (Figure 3C), our findings indicate that the pharmacological stimulation of β3-adrenergic receptors amplifies the increase in metabolic heat production observed during exposure to cool temperatures. We interpret these data to indicate that supraclavicular BAT has sufficient reserve to be further activated with mirabegron at these cool temperatures. This reserve could be attributable to β3-adrenergic receptor availability and/or increased recruitment of brown adipocytes, or alternatively, from the upregulation of the UCP-1 expression within active BAT depots (Dabrowska and Dudka, 2023). Whereas the use of infrared thermography and indirect calorimetry cannot distinguish between these factors, previous reports in humans using PET/CT have shown increases in BAT volume metabolic activity following β3-adrenergic stimulation (Cypess et al., 2015; Orava et al., 2011).

In humans, the cold-induced increase in the metabolic rate is attributed to the activation of shivering thermogenesis in skeletal muscle (Eyolfson et al., 2001; Haman and Blondin, 2017) and/or NST in both BAT and skeletal muscle (Blondin and Haman, 2018; Blondin et al., 2014). The increase in energy expenditure in our study was not accompanied by changes in shivering activity (i.e., MMG activity and bedside shivering scale; Figures 6A, B, respectively). Indices of supraclavicular BAT activation, however, were greater following the ingestion of 100 and 150 mg doses of mirabegron versus PLA (i.e., AUC for absolute and relative T_sc_; Figures 4C, I, respectively). As we did not observe shivering but found that T_sc_ was higher following mirabegron ingestion, we interpret these findings to suggest that the thermogenic effects of mirabegron are likely due to increased BAT activation, which is consistent with previous reports from other groups (Cypess et al., 2015; Loh et al., 2019). Despite this, NST in the skeletal muscle may have been an active contributor to our findings because its contribution to cold-induced thermogenesis has been reported by previous reports (Wijers et al., 2011; Blondin et al., 2017). The physiological mechanisms that underpin the skeletal muscle’s thermogenic contribution remain unclear, but they likely involve some combination of skeletal muscle uncoupled mitochondrial respiration and/or sarcoendoplasmic reticulum Ca^2+^ ATPase (SERCA) pump-mediated Ca^2+^ cycling (Blondin and Haman, 2018; Blondin et al., 2014).

Notably, the use of infrared thermography as a surrogate measure of BAT activation, while non-invasive, may not be as precise as other techniques such as PET/CT scans (Virtanen et al., 2009; Cypess et al., 2009; van Marken Lichtenbelt et al., 2009) or the use of magnetic resonance imaging (MRI) (Wu et al., 2020). However, the use of infrared thermography in our study was deemed appropriate because it allowed us to use a whole-body indirect room calorimeter to precisely assess metabolism and calculate the energy expenditure over 6 h of observation, which would be impractical for PET/CT or MRI. The high sensitivity of the whole-body indirect room calorimeter to detect the steady state and dynamic metabolic measurements (Baugh et al., 2023), combined with the non-invasive nature of infrared thermography, provided novel insight into the metabolic impact of BAT activation via β3-adrenergic receptor stimulation during prolonged exposure to a cool stress environment. The combination of whole-body indirect calorimetry with repeated infrared thermography has been suggested as a novel protocol for accessing BAT thermogenesis in resting humans (Van Schaik et al., 2023).

Despite the observed increases in energy expenditure that are likely attributed to BAT activation, mirabegron ingestion did not affect T_rec_ or the mean body temperature during the cool exposure compared to that in baseline or the PLA condition (Figures 5A, E, respectively). BAT plays an important role in the human metabolism (Cannon and Nedergaard, 2004), yet the extent to which it can contribute to the overall heat production during cold exposure remains debatable due to the volume of BAT available for activation in adult humans. Metabolically active amounts are reported to range from 63 g to 1 kg (Virtanen et al., 2009; van der Lans et al., 2013; Ouellet et al., 2011; Shin et al., 2014). These variations in detectable BAT volume are likely due to different experimental protocols along with individual variability (Ouellet et al., 2011). Thus, it is unclear whether there is a sufficient volume of BAT that could enhance temperature regulation during a cold exposure. Given the experimental conditions utilized in our study, it is unlikely that mirabegron-induced BAT activation would have increased T_rec_ or the mean skin temperature. However, it remains unclear whether mirabegron-induced BAT activation during more intense and/or prolonged cold exposure could influence thermal resiliency.

Participants reported higher thermal sensation ratings (e.g., felt warmer) following the 100 mg dose of mirabegron, aligning with the observed increase in the AUC for both absolute and relative T_sc_ (Figures 4C, I, respectively) for the 100 mg dose. Participants also generally reported less thermal discomfort (condition main effect *p* = 0.08), indicating that mirabegron ingestion may attenuate thermal discomfort. Thus, it appears as though mirabegron ingestion alleviates cold discomfort when given prior to exposure in a cool environment. Although our study was not directly powered to detect differences among the conditions for thermal perceptions, our findings are intriguing and warrant future studies that focus on thermal perceptions as the main outcome variables following mirabegron ingestion.

The ingestion of high doses of mirabegron (i.e., 100 mg–200 mg) also activates β1-adrenergic receptors, leading to the elevated heart rate, blood pressure, and myocardial oxygen demand (i.e., rate pressure product) in healthy individuals (Shin et al., 2014). In our study, we found that the mean heart rate during the interventions was higher following the ingestion of 100 and 200 mg of mirabegron versus PLA; however, these differences were nominal (100 mg dose: +5 bpm vs. PLA; 200 mg dose: +4 bpm vs. PLA). Although no significant differences in diastolic and the mean arterial blood pressure were observed (Figures 7E, G, respectively), we did find that the systolic blood pressure was higher following the ingestion of 150 and 200 mg doses of mirabegron versus PLA (Figure 7D). This observation is consistent with that observed by Loh et al., who observed a greater change in systolic blood pressure following the ingestion of 150 and 200 mg doses of mirabegron, but not the 100 mg dose, over a 3-h period in a thermoneutral environment (i.e., 24 °C–25 °C). Interestingly, although the systolic blood pressure was not higher following the ingestion of the 100 mg dose of mirabegron versus PLA, we found that all doses of mirabegron elicited a higher rate pressure product versus PLA (Figure 8D), suggesting augmented myocardial oxygen demand. Although such changes may be well tolerated in healthy individuals, they may pose risks for individuals with underlying cardiovascular disease, hypertension, or impaired autonomic regulation. Elevated myocardial workload in these populations could precipitate adverse events such as arrhythmias, ischemia, or hypertensive crises. Similar to our study, future studies that utilize high doses of mirabegron should consider the experimental environment and the health history of the participants to reduce the risk of an unanticipated cardiovascular event.

### Strengths and limitations

Our study offers several notable strengths and some limitations that require consideration. The use of a whole-body indirect calorimeter room provided a far more sensitive and reliable measure of whole-body energy expenditure compared to other methods of indirect calorimetry, such as metabolic carts (Baugh et al., 2023). As the primary function of BAT is to elicit thermogenesis, the use of the whole-body indirect calorimeter room was deemed essential as it provided precise quantification of the thermogenic potential of mirabegron during prolonged exposure to a cool environment. However, this prevented us from using PET/CT or MRI to quantify the BAT volume and activation. Despite this, infrared thermography has been shown to correlate well with ^18^F-fluorodeoxyglucose uptake measured by PET/CT scans (Law et al., 2018b) and has been previously utilized as an effective technique to detect cold-induced supraclavicular BAT activation (Ramage et al., 2016). In this context, we were able to capture supraclavicular BAT activation across multiple timepoints within the same and across different experimental visits without exposing the participants to ionizing radiation. We did not provide standardized meals in the days leading up to each study visit, limiting our ability to tightly control for macro- and micronutrient consumption, which may have impacted BAT activation (Noriega et al., 2023). However, the results of the 72-h dietary recall indicate that our participants did not significantly alter their dietary habits prior to the experimental visits. Due to the repeated measures study design and the half-life of mirabegron, we did not control for seasonality, which has been suggested to impact BAT activation (Labbe et al., 2016). Despite this, all the participants completed our study in less than 3 months, likely mitigating the influence of season on the participants.

Finally, our study did not control for the menstrual cycle phase in female participants, which has been suggested to impact BAT thermogenesis in healthy women (Fuller-Jackson et al., 2020). Recent evidence from the study by Fuller-Jackson et al. (2020) suggest that BAT activity is influenced by both sex and circulating sex steroid levels, with estrogen enhancing BAT thermogenic capacity, potentially via the upregulation of β-adrenergic receptor sensitivity. This finding supports the idea that fluctuations in endogenous hormone levels across the menstrual cycle may impact the magnitude of BAT activation. However, not controlling for the menstrual cycle phase in our study enhances its external validity, as it better reflects the real-world variation among women and provides insight regarding how mirabegron may influence thermogenesis under typical physiological conditions. Future investigations should seek to profile menstrual phase tracking to more precisely delineate the interaction between pharmacologic β3-adrenergic stimulation and the endogenous hormonal status.

### Perspectives and significance

Cold-acclimated (i.e., exposed to environmental stress in a controlled setting) individuals have shown greater reliance on BAT activation and attenuated dependence on shivering activity for cold-induced metabolic heat production compared to their pre-acclimatized state (van der Lans et al., 2013). Thus, the targeted stimulation of BAT could augment human thermal resiliency during exposure to cold environments. When behavioral thermoregulatory strategies (i.e., seeking shelter, wearing insulated clothing, and increasing physical activity) are not adequate and/or available during cold exposure, humans rely on physiological defense mechanisms such as cutaneous vasoconstriction (Charkoudian, 2003; Johnson and Kellogg, 2018) and increases in metabolic heat production (Blondin et al., 2014) to attenuate decreases in body temperature. Despite the effectiveness of these processes, prolonged cold exposure can significantly attenuate the functionality (i.e., manual dexterity and cognition) of those who are routinely asked to perform tasks in such environments (Gagge et al., 2011). Moreover, these individuals are at an increased risk of developing cold-related injuries (Imray et al., 2011), such as frostbite, hypothermia, and trench foot. These outcomes can lead to increased safety risks and productivity losses, emphasizing the necessity for alternative interventions. For this reason, the acute pharmaceutical augmentation of heat production, via mirabegron ingestion, is a possible method to enhance human resiliency in cold environments. The cool environmental conditions in our study were mild, and thus, we did not observe changes in T_rec_ or the mean body temperature following PLA or mirabegron ingestion. However, our data do indicate that supraclavicular BAT activation can be amplified during a cool exposure. Further work is required to elucidate the effectiveness of acute mirabegron ingestion to enhance human thermal resiliency to more extreme cold conditions.

Although our findings indicate that acute mirabegron ingestion augments energy expenditure during a cool environmental exposure, previous investigations have explored the effects of chronic mirabegron treatment on energy expenditure (O'Mara et al., 2020). Following a 28-day intervention of daily ingestion of 100 mg of mirabegron, O’Mara et al. (2020) observed an increase in baseline resting energy expenditure from day 1 to day 28 (+82 kcal/day), representing a 5.8% increase in daily energy expenditure from the pretreatment timepoint. This finding is of importance as it suggests that chronic mirabegron treatment promotes an increase in baseline energy expenditure that is sustained, likely through enhanced BAT activity as they observed increases in BAT volume. Thus, further work aimed at exploring the chronic thermogenetic effects of mirabegron supplementation may be of great relevance to individuals who are routinely exposed to extreme cold temperatures.

## Conclusion

In this study, we demonstrated that acute doses of mirabegron elicited greater energy expenditure during a prolonged exposure to a cool environment compared to PLA ingestion. We attribute the increase in thermogenesis following mirabegron ingestion to BAT activation, as the indices of supraclavicular BAT activation were elevated following mirabegron ingestion compared to PLA. Although we did not observe a change in T_rec_ or the mean body temperature during our study, our data indicate that β3-adrenergic receptor activation in BAT enhances thermogenesis in cool environments.

## Data Availability

The raw data supporting the conclusions of this article will be made available by the authors, without undue reservation.
